# Alginate as Dispersing Agent for Compounding Natural Rubber with High Loading Microfibrillated Cellulose

**DOI:** 10.3390/polym13030468

**Published:** 2021-02-01

**Authors:** Goragot Supanakorn, Nanthaphak Varatkowpairote, Siriporn Taokaew, Muenduen Phisalaphong

**Affiliations:** 1 Department of Chemical Engineering, Faculty of Engineering, Chulalongkorn University, Bangkok 10330, Thailand; gsupanakorn@gmail.com (G.S.); nanthaphak_wekar@hotmail.com (N.V.); 2 Department of Materials Science and Technology, School of Engineering, Nagaoka University of Technology, Nagaoka, Niigata 940-2188, Japan; t.siriporn@mst.nagaokaut.ac.jp

**Keywords:** natural rubber, microfibrillated cellulose, alginate, green composite, reinforcement

## Abstract

Natural rubber (NR) reinforced with high loading of microfibrillated cellulose (MFC) was fabricated in the presence of sodium alginate as a thickening and dispersing agent in NR latex. The tensile strength and Young’s moduli of the 50% wt. MFC loading-NR composites were 13.6 and 1085.7 MPa, which were about 11.3- and 329-times enhanced compared with those of the neat NR film. The maximum elongation at 313.3% was obtained from 30% MFC loading, which was a 3.3-fold increase of that of the NR film. The thermal stability of MFC–NR films was slightly reduced, while the glass transition temperature remained unchanged at −64 °C. The MFC–NR films exhibited high water adsorption ability, toluene resistance, and biodegradability.

## 1. Introduction

Natural rubber, a renewable thermoplastic polymer of 2-methyl 1,3-butadiene or isprene units (C_5_H_8_)n, is used in a numerous applications due to its superior physical and chemical properties, and excellent elasticity [1,2,3]. However, unmodified natural rubber has relatively low mechanical strength and lack of resistance to organic solvents [4,5]. To improve properties, NR products are usually modified by the vulcanization process. By an addition of sulfur and heat, linear chains of rubber are chemically cross-linked to form covalent bonds and consequently the vulcanized molecular network of rubber is strengthened [6,7]. However, this process has drawbacks regarding energy consumption in the production of rubber products. Biodegradation of vulcanized rubber material is also difficult due to the interlinkages of the poly(cis-1,4-isoprene) chains, resulting in low water absorption and low gas permeability of the material. Additionally, curing and anti-ageing agents in the vulcanized rubber products have antimicrobial effects. Unlike the chemical method, the reinforcement involving the use of fillers facilitates the improvement of properties in the form of composites [8]. Among fillers, carbon black is often of interest in the rubber industry to augment the toughness of the rubbery matrix. Carbon black is mainly produced by the incomplete combustion of heavy petroleum products, which is well-known to cause carcinogens and pollutants [9,10]. Silica is also widely used as rubber reinforcing filler; however, it has some disadvantages, including inefficient recovery and high density. A major drawback of using silica is its difficulty to process due to the polarity difference between silica and rubber [11]. Owing to the concerns of environmental protection and sustainability, the reinforcing materials in NR matrix should be environmentally friendly, biodegradable, and nontoxic. According to the many advantageous properties of natural cellulose fibers, such as good mechanical strength, wide availability, low density, low-price, and renewability [12], it is considered as a good candidate for NR reinforcing processes. Cellulose is a polysaccharide consisting of a linear chain of β-1,4 linked D-glucose units, which can be obtained from plants, algae, and bacteria. Cellulose microfibrils have an abundance of hydroxyl groups that are formed firmly through inter- and intra-hydrogen bonding leading to the high strength [13,14]. Hence, it has been used as green reinforcement filler for the different types of composites to achieve pronounced property improvements, especially, in terms of mechanical strengths. To reinforce the hydrophobic matrix of NR with hydrophilic compounds such as cellulose, the direct blending is limited due to the polarity difference between cellulose and NR, resulting in poor dispersion. Therefore, modifications of NR have been studied, for example, by epoxidation of NR to increase the interfacial compatibility between NR and cellulose before compounding [15]. On the other hands, attempts for cellulose modifications have also been made through complex modifications of cellulose by hydrophobization strategies such as surfactant adsorption [16], surface modification [17,18], and chemical grafting [19] in order to increase the surface hydrophobicity of the cellulose fibers. Furthermore, most studies generally use nanocelluloses or cellulose nanocrystal suspensions, which are less difficult in terms of forming uniform dispersion in NR latex [20,21,22,23]. Therefore, some extra steps to prepare nanocellulose before reinforcing into NR matrix are needed. 

This research purposes the simplified approach to reinforce microfibrillated cellulose (MFC) in NR matrix. Sodium alginate was used as a thickening agent to increase the mixture viscosity so as to retard sedimentation and increase the dispersion of the reinforcing filler in the NR matrix. Effects of MFC loading on the morphology, mechanical properties, thermal stability, solvent resistance, and biodegradability of NR composites were evaluated. 

## 2. Materials and Methods

### 2.1. Materials 

Natural rubber latex with 60% dry rubber content (60 phr) was supplied by the Rubber Research Institute of Thailand. Sheets of cellulose from eucalyptus pulp were kindly provided by the Teppattana Paper Mill Co., Ltd. (Bangkok, Thailand). The sheets were dried in an oven at 80 °C for 24 h to remove moisture prior to using in the experiment. MFC was prepared by crushing the dried cellulose sheet, grinding by using ball mill (PM 100, Haan, Germany), and sieving, respectively. After grinding, the size of MFC particle is in the range of 10–30 µm, with the average particle size of 26.5 µm (evaluated by using Laser particle size distribution (PSD)). Sodium alginate and other chemical reagents (toluene and DI water) were purchased from Sigma-Aldrich (Thailand) Co Ltd. (Bangkok, Thailand).

### 2.2. Preparation of MFC–NR Composite Films

MFC was dispersed in deionized (DI) water using ultrasonication at 500 W for 15 min prior to adding sodium alginate at 1% (*w*/*v*). Previously, sodium alginate at 1% (*w*/*v*) was found to be a suitable solution for good dispersion of fillers and the obtain MFC slurry could homogeneously blend with natural rubber latex (NRL) [24]. To fabricate composite films, 5 g of 60 phr NRL was slowly added to 30 mL of the MFC slurry. Then the mixture was stirred by high-frequency mechanical stirring until homogenous. After that, the films were cast in a plastic tray and dried in an oven at 40 °C for 24 h. The compositions of MFC and NR and labeling of the MFC–NR composite films are listed in Table 1.

### 2.3. Characterization of MFC–NR Composite Films

Morphologies and dispersion of MFC within the NR matrix were viewed by Field Emission Scanning Electron Microscopy (FESEM), model JSM-7610F (JEOL, Tokyo, Japan). The sample was prepared by quenching in liquid nitrogen, fracturing, and subsequently drying under vacuum condition. The surface and cross-section of samples were coated with a thin layer of gold. The energy of the accelerator beam was 2 kV. The maximum tensile strength, Young’s modulus, and elongation at break of NR composite films were investigated using Universal Testing Machine (Instron, Norwood, MA, USA) following the testing conditions reported in ASTM D882. The average values were determined from at least five specimens and reported as average values with standard deviation (SD). The crystallinity of the films was characterized using X-ray diffractometer (XRD, Bruker AXS Model D8 Discover, Germany) at 40 kV and 45 mA. The samples were scanned at 2θ of 5–40° at a step of 0.02°. Attenuated Total Reflectance Fourier Transform Infrared spectroscopy (ATR-FTIR) (PerkinElmer, Waltham, MA, USA) was used for detecting the functional groups of the MFC–NR composite films in the range of 400–4000 cm^−1^. The glass transition (T_g_) of composite films was evaluated by differential scanning calorimetry (DSC), NETZSCH 204 F1, (Selb, Germany). The samples were heated from −100 to 300 °C at a heating rate of 10 °C/min under nitrogen gas. Thermal decomposition was analyzed by thermogravimetric analysis (TGA), NETZSCH TG 209F3 (Selb, Germany) at the scanning temperature of 35–600 °C using a heating rate of 10 °C/min under nitrogen gas. The decomposition temperature (T_d_) of the sample at the mass loss of 5% was recorded. For water absorption capacity, the composite films (2.5 × 2.5 cm^2^) were immersed in DI water for 7 days under ambient condition. The swollen samples were removed from the water and excess water at the surface of the samples was blotted out by Kimwipes^®^ paper. The weight of wet sample was then weighed. All testing was carried out in triplicate. Water absorption of the sample was calculated by the following equation [24]:

$$\mathrm{Water}\;\mathrm{absorption}\;(\%)\;=\;\frac{W_{W\;}-\;W_{d}}{W_{d}}\;\times 100$$

where *W_W_* and *W_d_* denote the weight of the hydrated composite films saturated with DI water and the dry weight of the films before immersing in DI water, respectively. For the test of toluene uptake, dried sample films (2.5 × 2.5 cm^2^) were immersed in toluene at room temperature. The weight change was monitored at 1 h intervals for 8 h. The toluene uptake was calculated by the following equation [24]:

$$\mathrm{Toluene}\;\mathrm{uptake}\;(\%)\;=\;\frac{W_{T\;}-\;W_{d}}{W_{d}}\;\times 100$$

where *W_d_* is the initial dry weight of the sample and *W_T_* is the weight of the sample after immersion in toluene at time t. 

Biodegradation testing in soil was also carried out. The film sample (5 × 5 cm^2^) was dried until weight constant (*W*_0_), and afterwards buried in soil at the depth of 10 cm for 8 weeks under ambient condition. The experimental study of biodegradation in soil was performed as outdoor test under uncontrolled condition, where the temperature range was 24 to 35 °C. Every 2 weeks, the samples were removed from soil, washed with DI water, dried at 4 at 40 °C for 12 h and recorded for their weight (*Wt*). The weight loss was calculated by the following equation [25]:

$$\mathrm{Weight}\;\mathrm{loss}\;(\%)\;=\;\frac{W_{t\;}-\;W_{0}}{W_{0}}\;\times 100.$$

## 3. Results and Discussion

### 3.1. Morphology of the MFC–NR Composite Films

**Figure 1:**
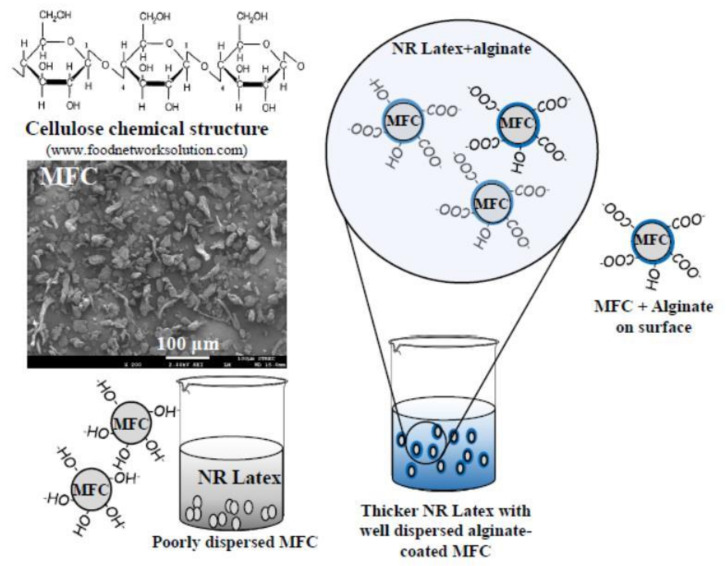
Schematic illustration of preparation of MFC–NR composites by latex compounding in the presence of alginate.

Under the visible observation in the mixture of MFC and NR latex without thickening/dispersing agent (alginate), MFC tended to agglomerate and be separated from NR latex due to the different polarities. However, by means of adding alginate in the MFC slurry prior to blending with NR latex, the homogeneous mixture of MFC and NR latex was observed, which confirms that alginate assisted the dispersion of MFC in NR latex. The water-soluble alginate, having an abundance of carboxyl and hydroxyl groups, could serve as a compatibilizer between hydrophilic MFC and hydrophobic NR by dissolution in water contained in the colloidal NR latex. Alginate could also form a thin film on cellulose fibers’ surface and prevent the agglomeration of cellulose in NL matrix leading to homogeneous suspension of water-insoluble MFCs in NR latex. The scheme of the proposed MFC–NR interface is illustrated in Figure 1.

**Figure 2:**
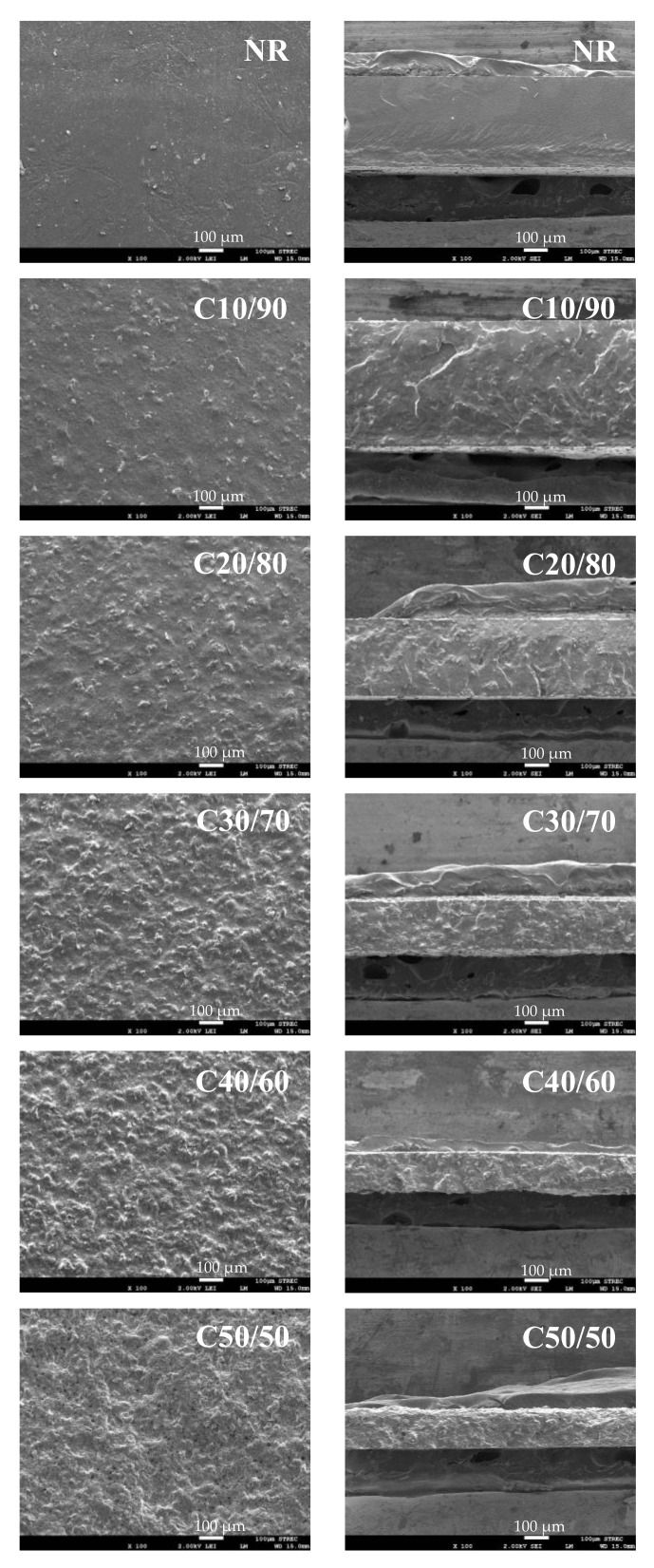
SEM images of surface (left) and cross-section (right) of NR and MFC–NR composite films.

SEM images of the NR composite films are shown in Figure 2. Dried NR film without loading of MFC appears as a smooth surface. For C10/90, C20/80, and C30/70 composites, MFCs were well distributed in NR matrices. At the higher MFC loading of 40 and 50%, the rougher surfaces and voids were noticed on the film surfaces. The cross-sectional morphologies indicated the homogeneity between NR and MFC/alginate. Sodium alginate was commonly used as a thickener agent to increase the viscosity of aqueous solutions. The mixture with high viscosity can retard sedimentation of fillers and can increase the dispersion of the filler in the NR matrix. Similarly, it was previously found that sodium alginate can be used as a thickening and dispersing agent for coal fly ashes as fillers in the NR matrix [24]. Additionally, the thickness of the composite films was decreased with higher MFC loading, which could be due to dense-packed cellulose formation through hydrogen bonding between abundant hydroxyl groups on cellulose chains [7].

### 3.2. Mechanical Properties

**Figure 3:**
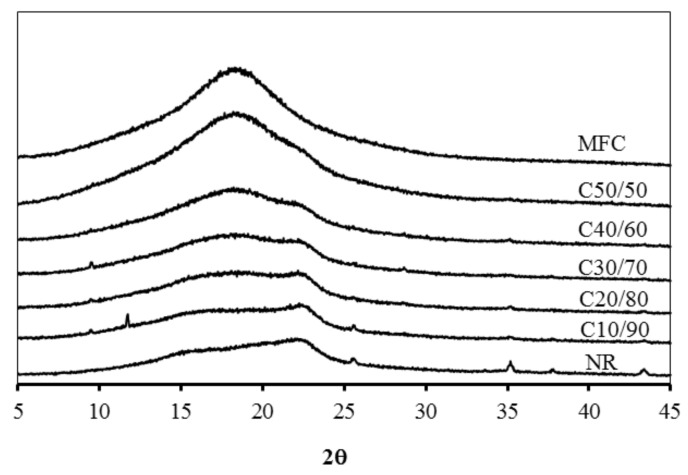
XRD patterns of MFC, MFC–NR composite, and NR films.

The mechanical properties in terms of tensile strength of NR were improved after reinforcement with MFC. As shown in Table 2, the unmodified NR film had low tensile strength of ~1 MPa and Young’s modulus of ~3 MPa. The tensile strength and Young’s moduli of the MFC–NR composites were increasingly enhanced proportional to the loading amount of MFC. Previously, the improved mechanical properties were reported in the relationship with crystallinity [26]. In this study, the increased tensile strength and Young’s modulus were in agreement with those of the degree of crystallinity (Cr) observed by XRD (Table 2). The composites became less amorphous with appearing crystalline broad signals of cellulose at 17–18° and 22–23° (Figure 3). At the maximum loading, C50/50 possessed remarkably improved tensile strength and exhibited Young’s modulus of 14 MPa and 1086 MPa, respectively, or about 11 and 329 times higher than those of the neat NR film. MFC at 10% loading in C10/90 film improved the elongation at break about twice from the neat NR having the elongation of ~70% (Table 2). The enhanced elongation of about four times was obtained from C20/80 and C30/70 films. This might be because of the blending with alginate, which could infiltrate the interface of NR and MFC phases, resulting in the improved flexibility of MFC–NR films. However, when MFC was loaded at 40 and 50%, the elongation values of C40/60 and C50/50 films drastically dropped to ~12 and 2%, respectively due to the relatively lower uniform dispersion of MFC in NR matrix at very high loading. Moreover, it is previously demonstrated that the strong interaction of hydrogen bonding from cellulose could cause the formation of a rigid three-dimensional network in the rubber matrix, leading to a robust structure, and a number of reinforcing filler-rubber polymer interactions could obstruct the mobility of the NR chain [1,4,22]. 

### 3.3. FTIR 

**Figure 4:**
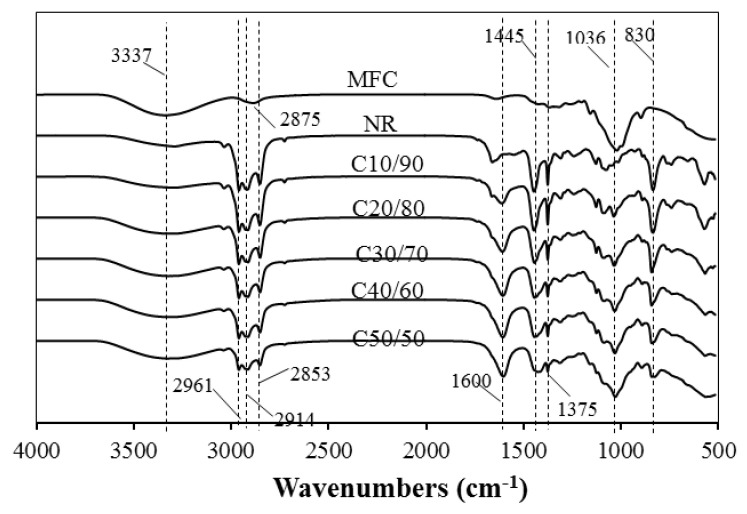
FTIR spectra of MFC, NR, and MFC–NR composites.

The chemistry and interaction between the highly polar MFC and the non-polar NR were analyzed using FTIR (Figure 4). The spectra of MFC show bands in the wide region of 3000–3500 cm^−1^ (centered at ~3337 cm^−1^), 2875 and 1019 cm^−1^, which were assigned to the stretching vibration of the OH group, C-H symmetric stretching, and C-O stretching vibration of cellulose, respectively [27]. The spectral for NR in the region of 3000–2700 cm^−1^ reveal peaks at 2962, 2914, and 2853 cm^−1^ corresponding to symmetric CH_3_, asymmetric CH_2_, and symmetric CH_2_ stretching vibrations, respectively. The sharp peaks of C=C stretching vibration, and CH_2_ and CH_3_ bending deformation of NR are shown at 1660, 1448, and 1376 cm^−1^, respectively. The rocking mode of CH_3_ also appears at 833 cm^−1^ [21,28]. 

For the FTIR spectra of the composite films, all the absorption bands which originated from MFC and NR were observed. The peaks of carboxyl and hydroxyl groups of alginate were found at ~3200 and 1600 cm^−1^, respectively. For C10/90 film, the effects of reinforcing MFC could be noticed by the stronger band of OH group and the occurrence of C-O stretching peak of cellulose at 1036 cm^−1^, which overlapped with a shoulder peak of C-C stretching vibration at 1040 cm^−1^ of NR. These bands, particularly in the OH stretching region, were more intense with increasing MFC content in the films, indicating induced hydrogen bonding. The increment of the hydrogen bonding of MFC could explain the results of the enhanced tensile strength and Young’s modulus [4,22]. On the other hand, the peaks around 1445, 1375, and 830 cm^−1^ became weaker when NR content was reduced. The intensity of peaks dominated by NR at 1660 and 1376 cm^−1^ belonging to C=C stretching and CH_3_ bending, respectively, became lower. The remarkable shifted peaks were not observed, suggesting that there were no chemical interactions between functional groups of MFC and NR. However, the weak interaction of cellulose fiber and alginate was previously reported [23].

### 3.4. Thermal Properties 

**Figure 5:**
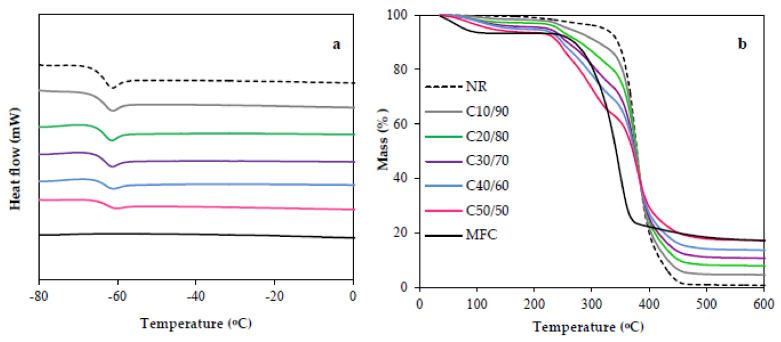
DSC thermograms (**a**) and TGA curves (**b**) of NR, MFC–NRs, and MFC.

Glass transition temperatures (T_g_) of MFC, NR, and MFC–NR films obtained from the endothermic peaks in DSC thermograms (Figure 5a) are listed in Table 2. The NR film became glassy at the temperature of −64.7 °C. For the composite films, range of T_g_ was quite similar (−63.4 to −64.5 °C) regardless the different composition of MFC and NR. Therefore, the reinforcement did not significantly affect the T_g_ as the consequence of the negligible chemical interaction between the MFC and NR segments confirmed by the FTIR result. The T_g_ values predominantly related to the nature of the polymeric matrix were reported [5,29]. 

The thermal degradation was determined by using thermogravimetric analysis (TGA). The TGA curves show % mass loss of MFC, NR, and MFC–NR composite films at various temperatures (Figure 5b). The peak of water evaporation from cellulose appeared around 100 °C. The MFC and NR decomposed in a single step with an initial decomposition of 251 and 348 °C, respectively. For MFC–NR composites, minor degradation started at ~250 °C, which contributed to the thermal degradation of alginate [30]. The degradation temperatures (T_d_) at the mass loss of 5% of MFC–NR films are given in Table 2. The T_d_ of MFC–NR films shifted to the lower temperature, which was influenced by MFC loading, because MFC has lower thermal decomposition relative to that of NR. The main decomposition pathway of cellulose related to oxygen atoms of hydroxyl functional group of cellulose facilitated the early thermal degradation [22]. Accordingly, the thermal stability of MFC–NR films was reduced as compared to NR film. At high MFC loading of 40–50%, the TGA curvature of C40/60 and C50/50 exhibited multiple degradation steps, which might be due to some agglomeration of MFC and lower uniform dispersion of MFC in the rubber matrix. At temperatures above 450 °C, the remained remaining weight (4–20%) of the decomposition products such as char, the non-volatile residual with high carbon content [1,22] after the pyrolysis, was found for MFC–NR films and the pure MFC film. The residual product at the end of the thermal composition related to the weight percentage of MFC added in the composite films. 

### 3.5. Water Absorption and Toluene Uptake

**Figure 6:**
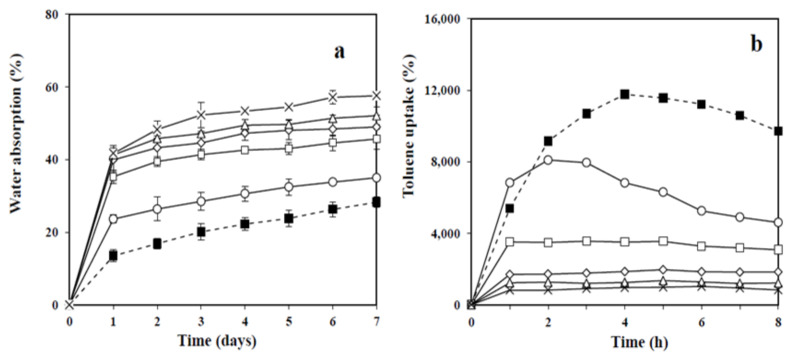
Water absorption (**a**) and Toluene uptake (**b**) of NR and MFC–NR composite films: NR (■); C10/90 (o); C20/80 (□); C30/70 (◊); C40/60 (∆); C50/50 (×).

Water absorption of MFC–NR films was carried out with respect to the immersion time for 0–7 days (Figure 6a). The reinforcement of MFC at 30–50%wt. within the NR matrix enhanced the rate of water absorption. During one day of immersion, water was rapidly absorbed into C20/80, C30/70, C40/60, and C50/50 films, whereas the neat NR and C10/90 films gradually absorbed water during entire 7 days. The degree of water absorption with time increased with increasing MFC content because of the favorable interaction between water and the hydroxyl group of cellulose. Consequently, water diffusion was improved in the composites of cellulose distributed NR matrix. The water absorption rate of all the samples was dropped significantly after a 1 day-immersion period in water. For this immersion duration, the composites containing 30–50% MFC yielded water absorption of 40–50% of its initial dry weight, with a gradual increase from the first day to the seventh day; whereas, C10/90 and NR films absorbed water at the maximum capacity of 35 and 28%, respectively.

As toluene is a common solvent for NR, the solvent uptakes of MFC–NR composites were determined by soaking them in toluene for 8 h. Figure 6b shows the very high toluene uptake rate of NR film during the first 4 h. The maximum uptake was almost 12,000% of its initial dry weight, and then the toluene uptake dropped due to the dissolution of NR in toluene. The reduction of toluene uptake was strongly influenced by loading cellulose, which could prevent the swelling of the polymer chain of NR [5]. Among the composites, C10/90 comprised the lowest weight percentage of MFC and exhibited a similar solvent uptake behavior to the pure NR film as a function of time, but the total toluene uptake of C10/90 film was decreased by a factor of ≈0.5 times of that of NR film. At a loading greater than 10% MFC, C20/80, C30/70, C40/60, and C50/50 slightly swelled at 0–2 h before reaching the equilibrium at less than 4000% toluene uptake. When content of cellulose in the composite films increased, swelling of the composite film in a nonpolar solvent decreased. The result in this study was in agreement with that of the previous study, of which nanocellulose reinforced NR rapidly swelled for 2 h before reaching equilibrium [5]. For this immersion duration, dissolution of the NR composites of C20/80, C30/70, C40/60, and C50/50 in toluene was not observed. The result showed that the structural stability and the degradation resistance of the NR composites reinforced with MFC in the non-polar solvent (toluene) were considerably improved. 

### 3.6. Biodegradation 

**Figure 7:**
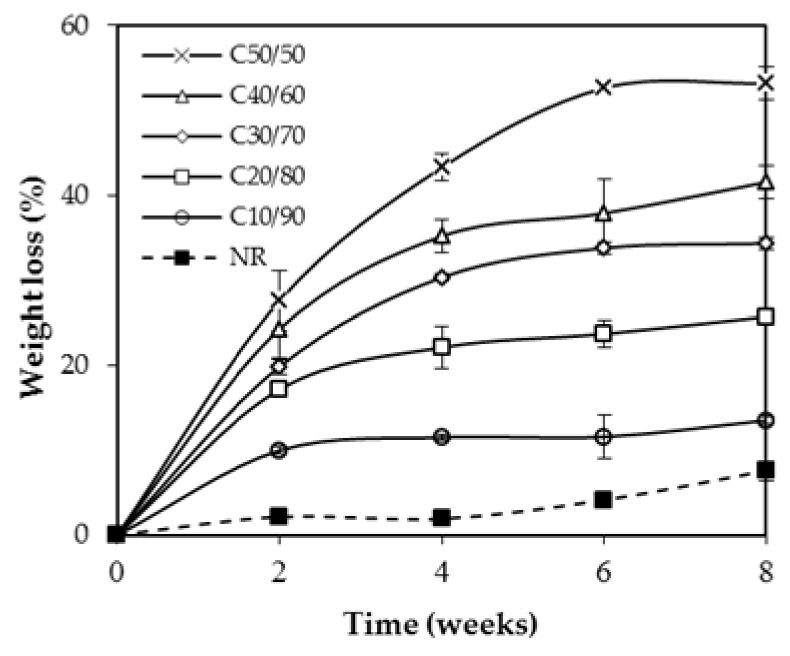
Biodegradation in soil of NR and MFC–NR composite films.

The waste of NR products, especially vulcanized NRs could cause a serious environmental concern in relation to their volume and low biodegradation rate [4]. The complex composition, structure, and additives of those NR products resulted in biodegradation resistance. In this study, the biodegradation in soil of MFC–NR films compared with the neat NR film was tested. Figure 7 shows the degradation rate of MFC–NR films in soil during 8 weeks of the experiment. The degradation of all films increased with incubation time and MFC loading. NR film showed a low degradation rate (less than 10% after 8 weeks). Cellulose appeared to enhance biodegradation [14,31,32]. The microbes from soil can exert cellulase cleaving the β-1,4 bond in cellulose chains capable of decomposing cellulose [33]. Consequently, the rate of degradation depended on the loading content of MFC. For C20/80, C30/70, and C40/60, the weight losses over 6 weeks of films in soil were ~24, 34 and 38%, respectively. The C50/50 film achieved the biodegradation at 45% after 4 weeks and more than 50% after 8 weeks. After cellulose fibers on the surface were rapidly degraded, the voids on the film surface allowed the greater mass of microorganisms in soil to colonize, attach, and secrete the depolymerase in the biodegradation process [34]. 

## 4. Conclusions

Based on the facile and green method by an addition of alginate as a thickening and dispersing agent, NR could be reinforced by MFC at the high loading concentration of 10–50%wt. The tensile strength and Young’s modulus of MFC–NR films were greatly improved with MFC loading content of 10–50%wt. Whereas, the elongation at break was improved at MFC loading concentration of 10–30%wt. The reinforcement showed no effect on the glass transition temperatures of MFC–NR films, but the thermal stability of MFC–NR films was decreased to some extent. The water absorption of MFC–NR films increased with increasing MFC loading content. The composite films exhibited improved structural stability in toluene. The composites were environmentally friendly, nontoxic, and biodegradable. According to green and facile procedures for the preparation with the use of cheap natural raw materials, the composite films of MFC–NR has a good potential to further develop as green products, such as sustainable packaging or biodegradable wound dressing. 

## Figures and Tables

**Table 1:** The composition of microfibrillated cellulose–natural rubber (MFC–NR) composite films.

Samples	MFC (g)	NR (g)
NR	0	100
C10/90	10	90
C20/80	20	80
C30/70	30	70
C40/60	40	60
C50/50	50	50

**Table 2:** Tensile strength (**σ_m_**), Young’s Modulus (E), elongation at break (**ε_b_**), degree of crystallinity (Cr), glass transition temperature (Tg), and decomposition temperature (Td) at mass loss of 5% of NR, MFC–NR composites, and MFC.

Samples	σ_m_ (MPa)	E (MPa)	ε_b_ (%)	Cr (%)	Tg (°C)	Td (°C)
NR	1.2 ± 0.1	3.3 ± 0.4	72.9 ± 4.0	0	−64.7	321.4
C10/90	1.8 ± 0.0	4.4 ± 0.1	147.4 ± 11.7	1.0	−64.7	259.6
C20/80	6.5 ± 0.2	9.9 ± 0.5	302.7 ± 1.2	3.4	−64.4	241.7
C30/70	10.4 ± 0.7	87.5 ± 4.9	313.3 ± 11.7	7.1	−64.2	223.1
C40/60	10.6 ± 0.3	528.7 ± 18.9	12.0 ± 1.0	10.6	−64.3	167.1
C50/50	13.6 ± 0.3	1085.7 ± 45.2	1.9 ± 0.1	14.6	−63.4	123.5
MFC	-	-	-	23.2	-	77.2
